# Assessment of foot-and-mouth disease risk areas in mainland China based spatial multi-criteria decision analysis

**DOI:** 10.1186/s12917-021-03084-5

**Published:** 2021-12-06

**Authors:** Wang Haoran, Xiao Jianhua, Ouyang Maolin, Gao Hongyan, Bie Jia, Gao Li, Gao Xiang, Wang Hongbin

**Affiliations:** Department of Veterinary Surgery, Northeast Agricultural University, Harbin, Heilongjiang 150030 PR China

**Keywords:** Foot-and-mouth disease, Multi-criteria decision analysis, Risk areas, Analytic hierarchy process

## Abstract

**Background:**

Foot-and-mouth disease (FMD) is a highly contagious viral disease of cloven-hoofed animals. As a transboundary animal disease, the prevention and control of FMD are important. This study was based on spatial multi-criteria decision analysis (MCDA) to assess FMD risk areas in mainland China. Ten risk factors were identified for constructing risk maps by scoring, and the analytic hierarchy process (AHP) was used to calculate the criteria weights of all factors. Different risk factors had different units and attributes, and fuzzy membership was used to standardize the risk factors. The weighted linear combination (WLC) and one-at-a-time (OAT) were used to obtain risk and uncertainty maps as well as to perform sensitivity analysis.

**Results:**

Four major risk areas were identified in mainland China, including western (parts of Xinjiang and Tibet), southern (parts of Yunnan, Guizhou, Guangxi, Sichuan and Guangdong), northern (parts of Gansu, Ningxia and Inner Mongolia), and eastern (parts of Hebei, Henan, Anhui, Jiangsu and Shandong). Spring is the main season for FMD outbreaks. Risk areas were associated with the distance to previous outbreak points, grazing areas and cattle density. Receiver operating characteristic (ROC) analysis indicated that the risk map had good predictive power (AUC=0.8634).

**Conclusions:**

These results can be used to delineate FMD risk areas in mainland China, and veterinary services can adopt the targeted preventive measures and control strategies.

**Supplementary Information:**

The online version contains supplementary material available at 10.1186/s12917-021-03084-5.

## Background

Foot-and-mouth disease (FMD) is an acute, febrile, highly contagious disease caused by the foot-and-mouth disease virus, infecting domestic and wild cloven-hoofed animals [1, 2]. The most common signs in infected animals are fever, blister formation and ulceration in the mouth, lower extremities and udders [3]. Globally, between 1995 and 2005, the number of FMD outbreaks reached an all-time maximum and have continued to be an important animal disease of economic concern [4]. The World Organization for Animal Health (OIE) classifies FMD as a notifiable animal infectious disease, and China classifies it as a Class I animal disease.

China is a major producer and consumer of meat products in the world. It is one of the countries more seriously affected by FMD [5]. The FMD virus isolates were first reported in China were in 1958 in Xinjiang Uyghur Autonomous Region (serotypes O and A) and Yunnan Province (serotype Asia1) of China [6, 7]. In 2005, the FMD virus was first detected in cattle in Wuxi, Jiangsu Province (serotype Asia 1). The disease spread rapidly in mainland China, causing severe economic losses in 17 provinces between 2005 and 2009 [8]. Cattle are the main animals infected by serotype Asia 1. Due to the efforts of the government and farmers, FMD virus (serotype Asia 1) did not appear in mainland China after 2009. Since 2010, FMD virus (serotypes O and A) have been endemic and sporadic in many provinces, mainly in northwestern and southeastern China [9]. According to official data, 140 outbreaks have occurred in China since 2010. In October 2020, a total of 70 cattle and 6 cattle were affected in Heshuo County, Xinjiang Uyghur Autonomous Region. Xinjiang and Tibet are the provinces with the highest outbreaks of FMD in mainland China, which may be related to the high population density of susceptible species in the regions [9]. Also, the farming pattern in these regions is mostly small households and grazing, which may increase the risk of outbreaks.

Risk factors associated with FMD outbreaks have been reported in previous studies, including climatic factors, livestock density, transportation and breeding factors [10–12]. Given the considerable impact of FMD, a significant number of studies focused on evaluating risks, which have sought to provide guidance it mitigates risk, conduct contingency planning, and insights to potential economic outcomes [13]. The majority of this work has likely resulted from OIE requirements to conduct risk and economic consequence assessments, resulting in most available assessments being narrowly focused on specific geographic regions and often addressing OIE guidelines [14]. To avoid inaccuracy of assessment, countries have conducted FMD risk assessments based on local prevalence and risk factors, including Thailand and Brazil [15, 16].

Multi-criteria decision analysis (MCDA) is a mathematical approach that provides great value in decision systems [17]. By combining spatial geographic data and its weighted, it is converted into a decision map [18]. It has a wide range of applications, mainly in environmental studies, land suitability assessment and epidemiological risk area assessment [19–21]. In health geography, this approach can reflect the complexity of spatial risk factors that affect the disease occurrence and consider multiple criteria for better understanding of disease characteristics [18]. For example, Sangrat et al. modeled FMD risk areas in Thailand based on MCDA and showed that some regions in western, central, upper northern, northeastern, and southern Thailand were identified as risk areas for FMD, which were associated with a large number of previous FMD epidemics, distribution of livestock markets, and high density of cattle [15]. Weerapong et al. identified three types of spatial risk factors for Nipah virus transmission in pig farms through expert assessment, which included natural host factors, intermediate host factors, and environmental factors, and the results indicated that risk areas were mainly concentrated around bat colonies [21]. Alimi et al. evaluated the risk areas for Malaria epidemics in northern South America, and the results showed that areas along rivers and coasts have higher risk than other areas [22].

The aim of this study is to assess the risk areas of foot-and-mouth disease in mainland China based on MCDA. The results of this study will support disease control measures and implementation of risk-based surveillance strategies.

## Results

### Prevalence of FMD in mainland China from 2010 to 2020

**Fig. 1:**
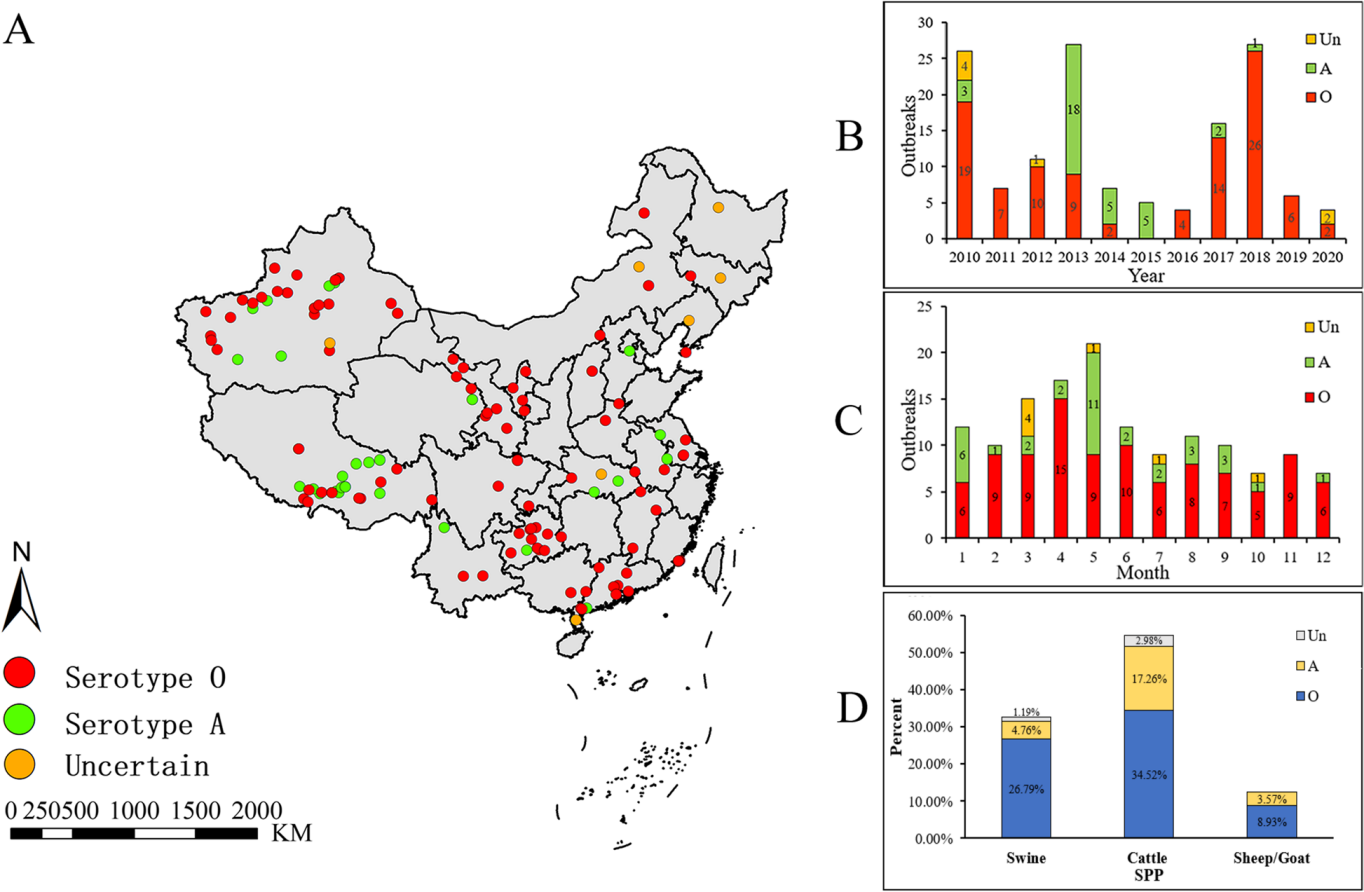
The prevalence of FMD in mainland China from 2010 to 2020. **A** Geographical distribution. **B** Annual outbreaks. **C** Monthly outbreaks. **D** The proportion of species affected. Uncertain: an outbreak of FMD at this location, but its serotype was not reported

In this study, a total of 140 FMD outbreaks were recorded between 2010 and 2020, including Serotype O (99), Serotype A (34) and uncertain Serotype (7). The outbreak was mainly distributed in the western and south-central of mainland China, including Xinjiang, Tibet, and Guizhou (Fig. 1A). Most outbreaks of FMD occurred in 2010 and 2018, with the main prevalent Serotype O (Fig. 1B). For the season, spring (March, April, May) was the season of high FMD prevalence (Fig. 1C). Of all domestic animals, cattle were the main susceptible animals (54.76%), followed by pigs (32.74%), and goats and sheep (12.50%) (Fig. 1D).

**Fig. 2:**
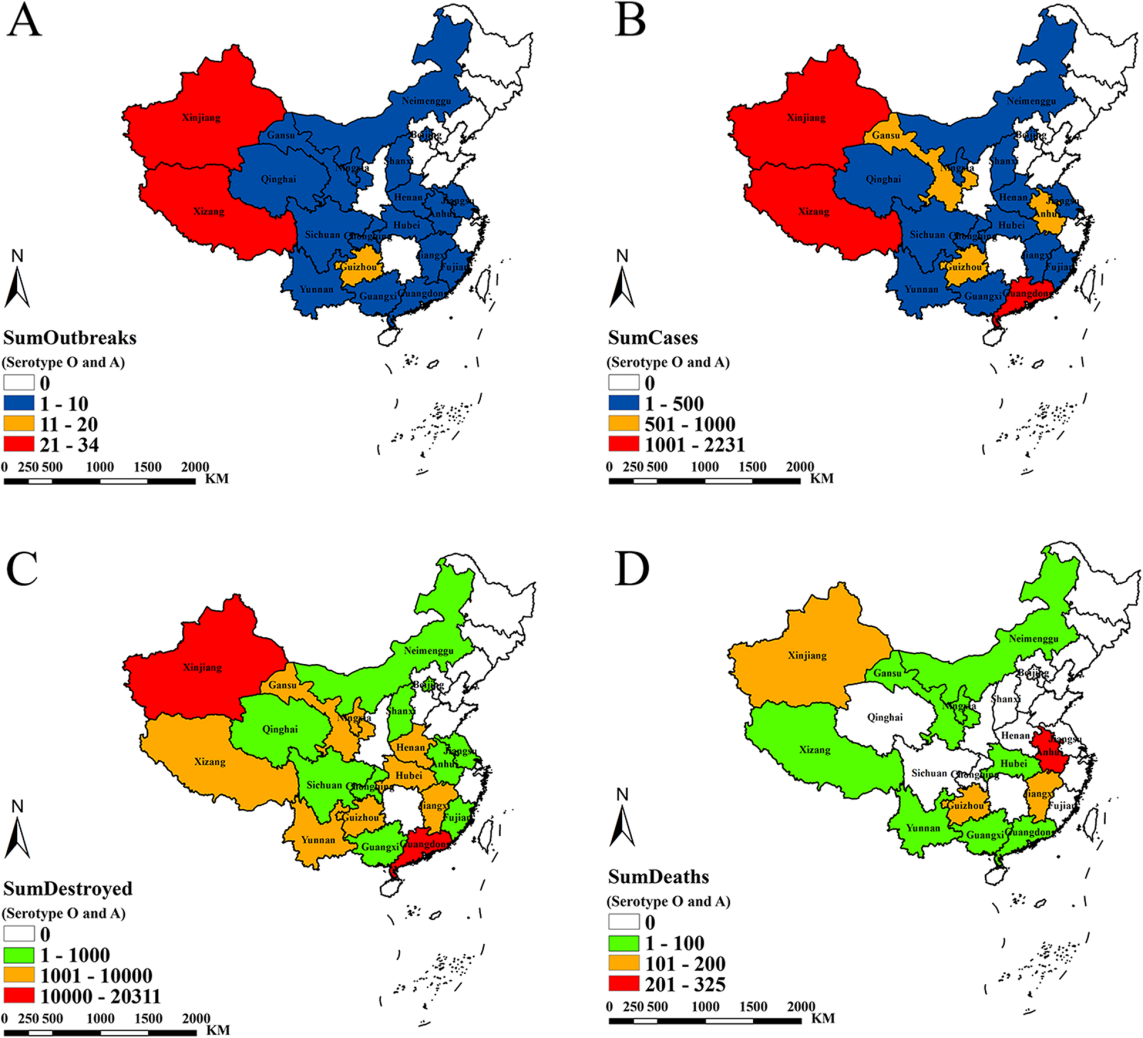
Spatial distribution of FMD in mainland China from 2010 to 2020. **A** Number of FMD outbreaks. **B** Number of FMD cases. **C** Number of FMD destroyed. **D** Number of FMD deaths

Between 2010 and 2020, twenty-one provinces in mainland China were affected by FMD in varying degrees. The sum-outbreaks, sum-cases and sum-destroyed in every province showed that the western of mainland China was severely affected because the sum-outbreaks, sum-cases and sum-destroyed were higher than other provinces (Fig. 2A-C). The sum-deaths were relatively low due to the aggressive control measures taken by the government and farmers, preventing large-scale livestock deaths (Fig. 2D).

### Risk factors weight

Ten important risk factors, such as buffalo density, cattle density, pig density, goat density, sheep density, distance to previous outbreak points, distance to national boundaries, distance to livestock market and slaughterhouse, major road density and grazing area were identified (Table 1). Distance to previous outbreak points, grazing area, and cattle density were considered as the most important risk factors for FMD outbreaks.

**Table 1:** The standardization and weight of risk factors for risk mapping

Risk factors	Average weight	Standard deviation	Standardization	Source	Reference
Buffalo density (head/km^2^)	0.12	0.0219	Sigmoidal increase	FAO	[11]
Cattle density (head/km^2^)	0.16	0.0282	Sigmoidal increase	FAO	[11]
Pig density (head/km^2^)	0.08	0.0063	Sigmoidal increase	FAO	[23]
Sheep density (head/km^2^)	0.04	0.0141	Sigmoidal increase	FAO	Self assessment
Goat density (head/km^2^)	0.04	0.0089	Sigmoidal increase	FAO	[11]
Distance to previous outbreak points (Km)	0.18	0.0282	Sigmoidal decrease	FAO	[24]
Distance to national boundaries (Km)	0.08	0.0179	Sigmoidal decrease	NGCC	[23]
Distance to livestock market and slaughterhouse (Km)	0.10	0.0155	Sigmoidal decrease	AMAP	[25]
Major road density (Km)	0.06	0.0190	Sigmoidal decrease	NGCC	[26]
Grazing area (Km)	0.14	0.0276	Sigmoidal increase	LUH	[27]

*FAO* The Food and Agriculture Organization, *NGCC* National Geomatics Center of China, *LCH* Land-Use Harmonization

### Risk map of FMD in mainland China

**Fig. 3:**
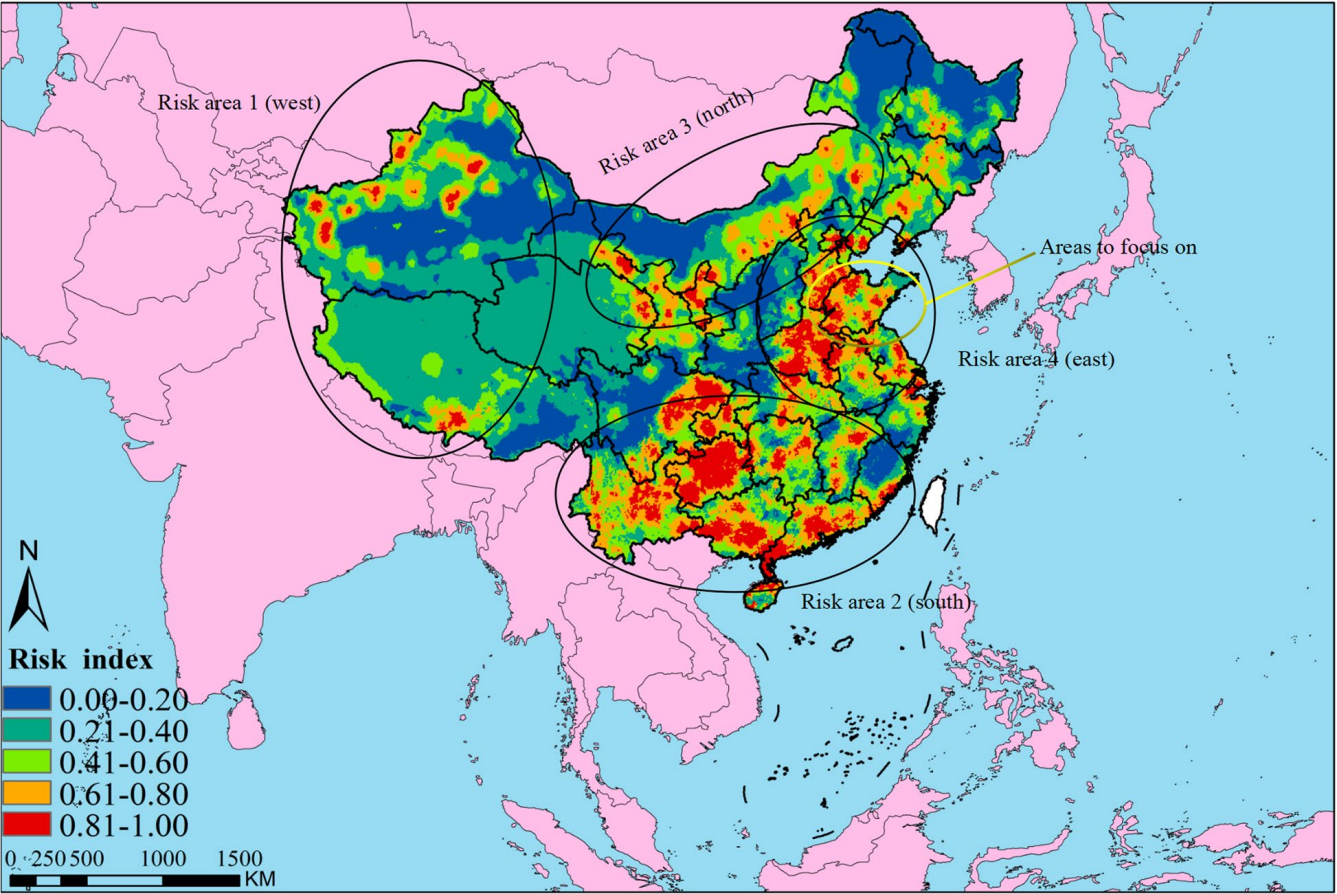
Risk map of FMD in mainland China

Figure 3 shows the risk map of FMD in mainland China. Four major risk areas were identified in mainland China, including western (parts of Xinjiang and Tibet), southern (parts of Yunnan, Guizhou, Guangxi, Sichuan and Guangdong), northern (parts of Gansu, Ningxia and Inner Mongolia), and eastern (parts of Hebei, Henan, Anhui, Jiangsu and Shandong). We assessed a risk province (Shandong) where FMD had never occurred.

### Uncertainty map

**Fig. 4:**
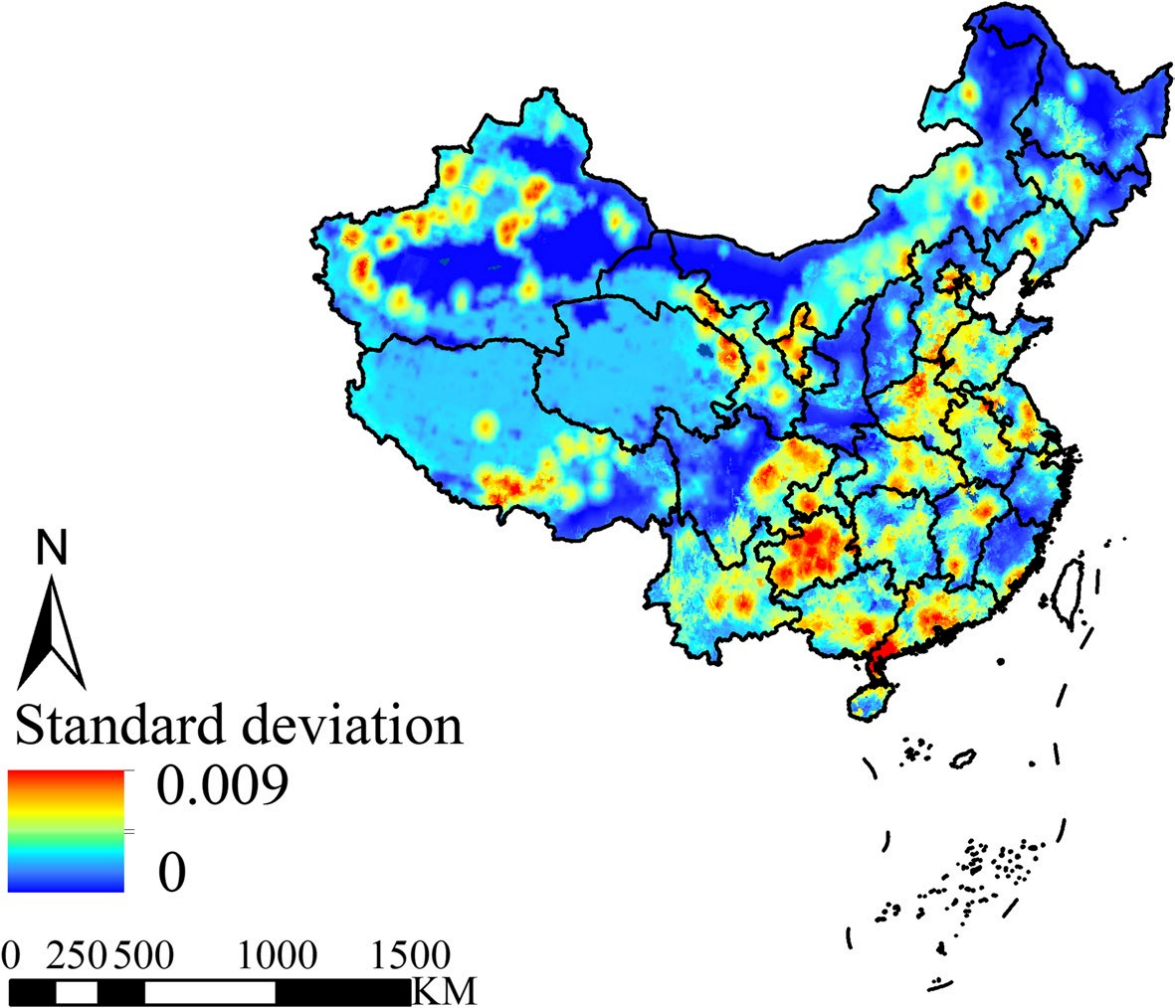
Uncertainty map (The maximum standard deviation of 4000 adjusted-weight risk map)

The uncertainty map represents the maximum standard deviation of the 4000 adjusted-weight risk maps (Fig. 4). The FMD risk map predicted based on risk factors was robust, with a maximum standard deviation less than 0.01, indicating that the predictive ability of the risk map is stable when the weights of risk factors are changed. The uncertainty map highlighted spatial heterogeneity, with higher uncertainty in high-risk FMD regions.

### Sensitivity analysis

**Fig. 5:**
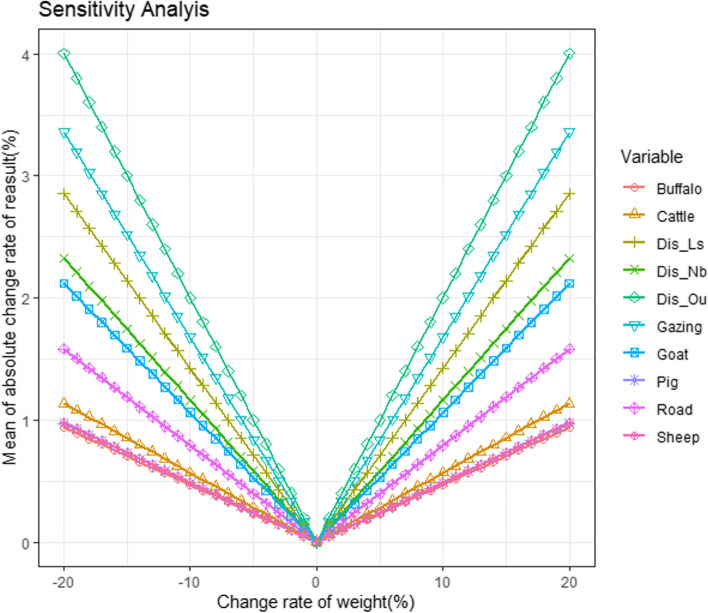
Mean absolute values of the change rate (MACRs) for the risk maps under simulations. (Buffalo: buffalo density; Cattle: cattle density; Dis_Ls: distance to livestock market and slaughterhouse; Dis_Nb: distance to national boundaries; Dis_Ou: distance to previous outbreak points; Gazing: grazing area; Goat: goat density; Pig: pig density; Road: major road density; Sheep: sheep density.)

As shown in Fig. 5, the MACRs showed an approximately linear increase with the absolute value of the change rate of the weights. The MACRs were the same for the same change factor when the absolute value of the change rate of the weights was the same. The same factor weight increased or decreased by the same value, and its sensitivity to the results was the same. The most sensitive factors were distance to previous outbreak points, followed by grazing area, distance to livestock market and slaughterhouse, distance to the national boundaries, goat density, major road density, cattle density, sheep density, pig density and buffalo density. Distance to previous outbreak points showed the highest sensitivity to weight changes, with the highest MACRs (4.4916%), which is much lower than the corresponding weight change rate (20%), indicating that the assessment of the FMD risk map is relatively stable.

### Risk map validation

**Fig. 6:**
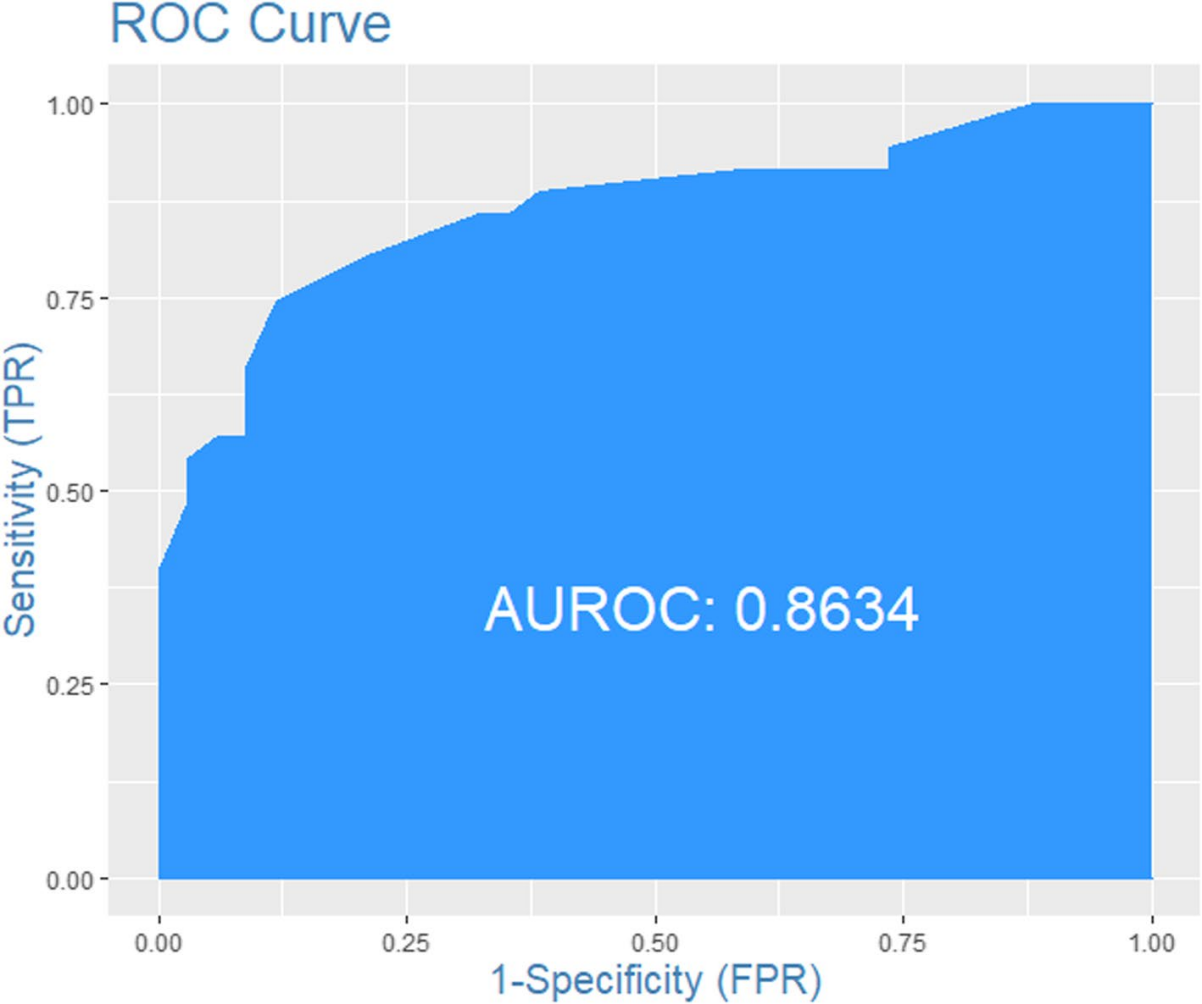
ROC of risk map

A total of thirty-five FMD outbreaks from 2018 to 2020 were used for model validation. Most of the outbreak points were found in the high-risk areas of the risk map. ROC results also showed that the prediction capacity of the risk map was good (AUC = 0.8634, Fig. 6).

## Discussion

Due to the prevalence of FMD in China, only some literature have explored the history and viral characteristics of outbreaks through phylogenetic analysis, and the risk of entering China through animal migration through geographic information systems, risk assessment and epidemiological models [28]. However, studies assessing risk areas in China are scarce. This study constructed an FMD risk map in mainland China-based on spatial MCDA.

Four major risk areas were identified in mainland China, including western (parts of Xinjiang and Tibet), southern (parts of Yunnan, Guizhou, Guangxi, Sichuan and Guangdong), northern (parts of Gansu, Ningxia and Inner Mongolia), and eastern (parts of Hebei, Henan, Anhui, Jiangsu and Shandong). We assessed a risk province (Shandong) where FMD had never occurred. Therefore, it is necessary to take measures to prevent outbreaks of FMD in mainland China. These areas are different in geographic location, climatic conditions, transportation, trade and livestock feeding management. In this study we presented some recommendations based on the characteristics of each region.

Western mainland China (parts of Tibet and Xinjiang), has always been a high outbreak area of FMD. China shares its borders with thirteen countries. Location may face the risk of FMD epidemic caused by cross-border transportation of wild animals or illegal livestock in other countries. Elnekave's research results showed that the risk of FMD infection in livestock in areas near the border was relatively high [29]. The highlands, mountains and basins are the dominant geographical features, which have made grazing the dominant livestock rearing and management practice in the region. Grazing is done in areas close to borders, leading to encounters with other FMD-affected livestock and wildlife, whose common behavior may increase the risk of disease transmission [27]. Previous studies reported the aggregation of different animals at the same watering points and pastures as a cause of FMD transmission and persistence [30]. However, the risk of epidemics exists in natural rearing and farmers captive breeding methods, even if farmers do not regularly practice grazing. Mixed rearing of cattle, sheep and goats is one of the risk factors for FMD outbreaks and transmission [31]. Balinda’s investigation concluded that the prevalence of FMD in small ruminants in Uganda was associated with outbreaks in cattle [32]. This may be related to the fact that small ruminants exhibit less severe clinical signs of FMD and are usually not on vaccination schedules [33]. The outbreaks that occurred in Tibet in 2013 were distributed along the main traffic routes, reflecting the association of animal movement with the occurrence of outbreaks [34]. The combination of grazing, mixed feeding, animal movement and livestock trade with border countries increases the probability of FMD outbreaks in these areas. These aspects, improvement of herders’ knowledge of the disease and increase of vaccination rates should be considered for the prevention of FMD.

During 2010-2020, we found a higher outbreak in spring. According to Guerrini’s study showed that the season affects the timing of FMD outbreaks across regions [35]. The reason for the outbreak may be that the climate has just warmed up and farmers need to cultivate and graze, which increases the risk of FMD for livestock. These conditions also apply to northern mainland China, including Gansu, Ningxia and Inner Mongolia. Although these regions have different climatic conditions, the northern region with large grasslands and adjacent borders is also dominated by grazing as a feeding management practice.

For the eastern and southern parts of mainland China, small-scale farming is the dominant feeding practice [36]. Animals are usually kept in free-range grazing systems and open barns, which are managed in a simpler way. Vaccination is the most reported preventive measure in surveys of small-scale farms [37]. So necessary vaccination programs and vaccine effectiveness are key to preventing outbreaks of FMD on small farms. Some farmers mistakenly identify FMD as stomatitis, a mucosal disease. Therefore, it is extremely important for small-scale farmers to improve their knowledge and understanding of FMD and the role of the vaccine. In the risk map, the complex transportation and aggregated livestock in the south had a great weight in the risk assessment [23, 26]. Human transmission by mechanical transfer or transportation is a factor that is not controlled easily. The government needs to strengthen regulation during animal import and export, regular spot checks and monitoring of livestock trading markets and slaughterhouses [25].

Shandong was assessed as a risk area, where no FMD was observed. There were some errors in the assessment with empirical data. But, considering the risk factors in Shandong, we believe that need to keep an eye on this province in the future. Outbreaks of FMD in nearby regions are likely to increase the risk of epidemics [24]. Moreover, there are some limitations to this study. For example, more risk factors need to be considered when constructing risk maps. More experienced experts need to be involved in assessing the importance of risk factors. Some subjective factors in scoring the risk factors may lead to some deviations from the actual situation. Therefore, in future research, we will combine subjective experience and objective factors to improve the accuracy of prediction results.

In this study, we assessed the FMD risk areas in mainland China-based on spatial MCDA and identified four major risk areas. Spring was the main season for FMD outbreaks in China. Risk areas were related to distance to previous outbreak points, grazing areas, and cattle density. These results can be used to delineate FMD risk areas in China and take target preventive measures and control strategies.

## Conclusion

This study was based on spatial MCDA to assess FMD risk areas in mainland China. Four major risk areas were identified in mainland China, including western (parts of Xinjiang and Tibet), southern (parts of Yunnan, Guizhou, Guangxi, Sichuan and Guangdong), northern (parts of Gansu, Ningxia and Inner Mongolia), and eastern (parts of Hebei, Henan, Anhui, Jiangsu and Shandong). Risk areas were associated with the distance to previous outbreak points, grazing areas and cattle density. ROC analysis indicated that the risk map had good predictive power (AUC=0.8634). These results can be used to delineate FMD risk areas in mainland China, and veterinary services can adopt the targeted preventive measures and control strategies.

## Materials and methods

### Study framework

**Fig. 7:**
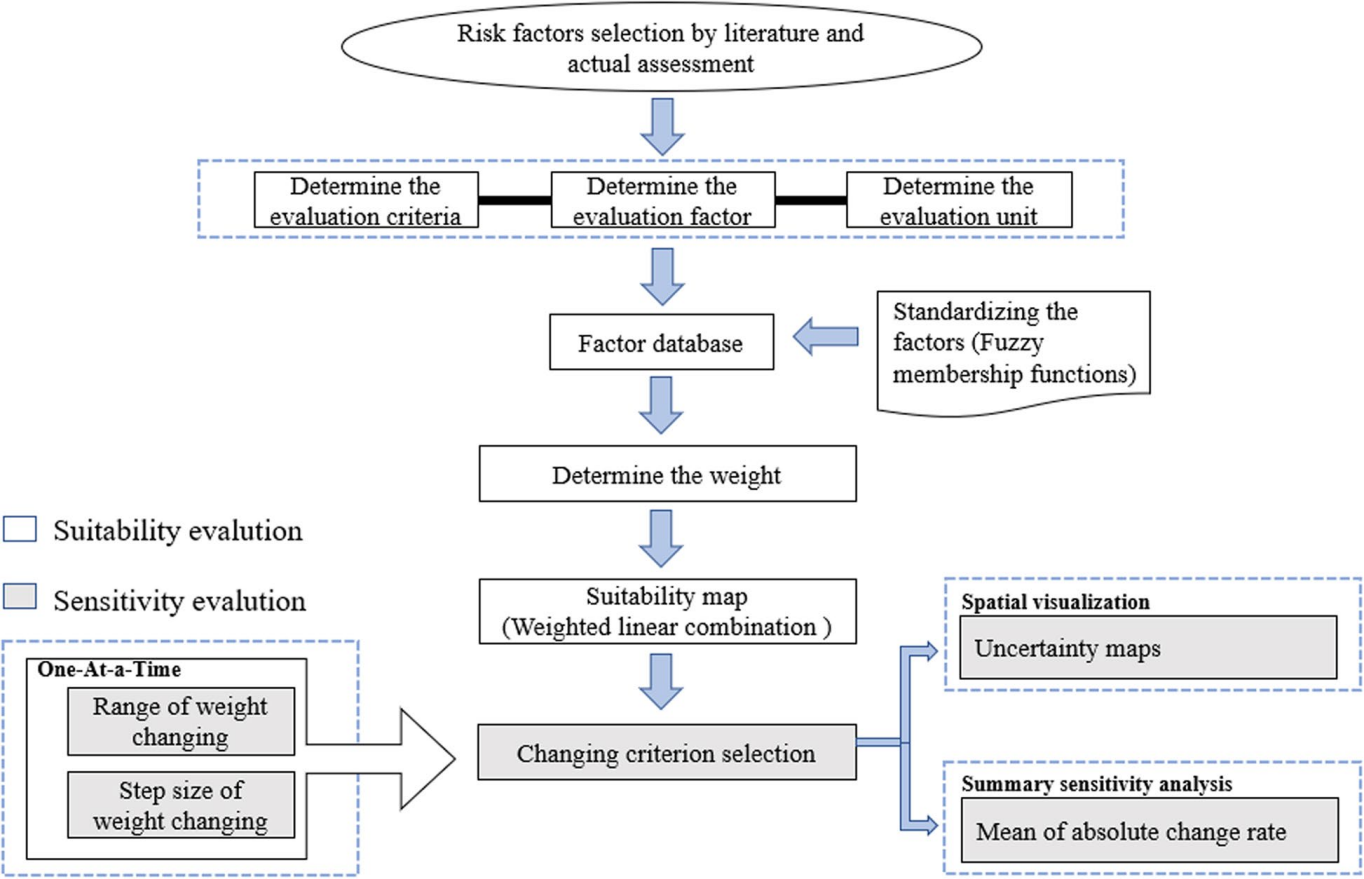
Study framework of FMD risk areas in mainland China based on spatial MCDA

In this study, MCDA was applied to assess the risk areas for FMD in China based on FMD outbreak data between 2010 and 2020 (Fig. 7). The data of outbreaks were from the Ministry of Agriculture of the People’s Republic of China (MAPRC) and the Food and Agriculture Organization of the United Nations (FAO).

1. We used Web of Science, PubMed and Google Scholar to select and evaluate the risk factors associated with the occurrence and spread of FMD. The five-Likert scale is used to select the ten most important spatial risk factors.
2. Analytic hierarchical process (AHP) was applied to assess the weights occupied by ten risk factors in FMD outbreaks.
3. Weighted linear combination (WLC) was used to transform the empirical data into risk map after standardization of all risk factors.
4. Sensitivity and uncertainty analysis was performed on the risk map.

### Data transformation and standardization

We selected fifty risk factors from the literature and self-assessment (Supplement 1), these factors were obtained from actual surveys and analyses. The selected risk factors were classified and scored using the five-Likert scale [15]. Ten professional veterinarians participated in this work, including three Ph.D. and seven M.S. Ten most important risk factors for FMD outbreaks were selected, including livestock distribution, environment, transportation and location conditions.

Density data for buffalo, cattle, pigs, goats and sheep were collected to the extent of mainland China, and resampling was applied to transform them into map layers with a spatial resolution of 2.5 arcmin (approx 5 km × 5 km). Euclidean distances were used to calculate distances to previous outbreak points, national borders, livestock markets and slaughterhouses, with the risk area limited to 100 KM [38]. The data were transformed into map layers with distances in km to each location. The kernel density estimation (KDE) was used to calculate densities of major railroads and roads in mainland China. Complex distance was used to measure local density changes in this method.

In this study, five types of relationships were proposed (linear, increasing sigmoidal, decreasing sigmoidal, symmetrical, and bi-directional). Based on previous literature for the relationship between these risk factors and FMD, sigmoidal was finally determined [15]. Decreasing sigmoidal was used in fuzzy affiliations for distance to previous outbreak points, distance to national boundaries and distance to livestock market and slaughterhouse. Increasing sigmoidal was used in other risk factors. After standardizing all risk factors, AHP was used to determine the weights. Distance to previous outbreak points, grazing area, and cattle density were considered as the most important risk factors for FMD outbreaks. Standardized spatial layer maps of all risk factors were calculated by fuzzy membership functions at a scale of 0–1 (unsuitable to perfectly suitable, Supplement 2).

All geographic data were calculated and transformed using ArcGIS 10.2 (ESRI, Redlands, CA, USA). The standardization of the risk maps was performed using IDRISI (Clark Labs, Worcester, USA).

### Risk factors weight

AHP was used to determine the weights of risk factors. The hierarchical structure of FMD risk areas assessment was divided into a target layer, a criterion layer, and an index layer. The factors were grouped according to their characteristics. The elements of the same level were dominated by the elements of the previous level. It dominated certain elements of the next level (Supplement 3). The judgment matrix was constructed by comparing the importance of all factors at each level using a 9-level scale. “1” means that the two elements are of equal importance; “3” means that the former is moderately important than the latter; “5” means that the former is strongly important than the latter; “7” means that the former is very strongly important than the latter; “9” indicates that the former is extremely important than the latter; 2, 4, 6 and 8 are the intermediate values of the above judgments. For weight calculation and hierarchical single ranking, we applied the square root to calculate the eigenvectors and eigenvalues. The resulting eigenvectors are the weight ranking of the factors. Equation 1 is given as follows:

1 $${\displaystyle \begin{array}{c}{M}_i={\prod}_{i=1}^n{P}_{ij}\kern0.5em \left(i,j=1,2\dots n\right)\\ {}\varpi =\sqrt[n]{M_i}\\ {}w={\left({w}_1,{w}_2,\dots {w}_n\right)}^T\\ {}\lambda \kern0.5em \mathit{\max}={\sum}_{i=1}^n\frac{(PW)i}{nw_i}\end{array}}$$

Where Mi is the product of the elements in the i-th row in the judgment matrix P; $\overline{\omega\ }$is the geometric mean of Mi; Wi is the weight of the i-th factor; W is the eigenvector; λmax is the maximum eigenvalue. The judgment matrix was constructed by comparing the scores of two elements. If the scores of each two elements are objectively consistent, the whole judgment matrix must be completely consistent. The essence of the consistency test is to verify the reasonableness of the constructed judgment matrix. Equation 2 is given as follows:

2 $${\displaystyle \begin{array}{l} CI=\frac{\lambda \kern0.5em \mathit{\max}-n}{n-1}\\ {} CR=\frac{CI}{RI}\end{array}}$$

λmax is the maximum eigenvalue; CI is the consistency index. If CI = 0, there is complete agreement; CI close to 0, there is satisfactory agreement; The larger the CI, the more serious the inconsistency. The RI is the average random consistency index (Table 2 showed the value of RI). Considering that the deviation of consistency may be due to random reasons. Therefore, when testing whether the judgment matrix is consistent, it is also necessary to compare CI and RI to derive the consistency ratio (CR).

**Table 2:** Standard value of RI (The standard is different, the value of RI will also have a slight difference)

n	1	2	3	4	5	6	7	8	9	10
RI	0	0	0.52	0.89	1.12	1.26	1.36	1.41	1.46	1.49

CR < 0.10 indicates a high level of consistency in the pairwise comparisons. However, if CR ≥ 0.10, the original values in the pairwise comparison matrix should be reconsidered and revised. R software version 4.0.3 (R Foundation for Statistical Computing, Vienna, Austria) was used for the application of AHP, and we provided the R code in this study (Supplement 4).

### Risk map

A risk map was produced from the selected spatial risk factor layers and weighted using WLC. Equation 3 is given as follows:

3 $$R={\sum}_{i=1}^n{w}_i\kern0.5em \nu \left({a}_i\right)$$

Where n is the number of risk factors; w is the weight of factor I; v is the value function of risk factors at level i(a_i_). R is the total value of risk factors at each cell level in the end [39]. ArcGIS 10.2 was used to map and classify the risk map.

### Sensitivity analysis and uncertainty

The one-at-a-time (OAT) was used to conduct sensitivity analysis. The change in the weight value of only one factor at a time (other factors remain unchanged as much as possible) reflects the degree of influence and regularity of the single factor weight change for results. For the adjustment weight (wa) of each main factor, the rate of change was between -20 and 20%, and the step size was 1%. The weights of other factors (wi) were calculated as follows (Eq. 4):

4 $$wi=\left(1- wa\right)\ast \frac{Wi_0}{1-{wa}_0}\kern0.5em 1\le i\le n,i\ne a$$

Where Wi_0_ is the initial weight of each risk factor; Wa_0_ is the initial weight of the main change risk factor. The sum of all weights is equal to 1 [40].

The mean of absolute change rates (MACRs) of the adjusted-weight risk maps were calculated as follows (Eq. 5):

5 $$MACRs={\sum}_{k=1}^N\frac{1}{N}\ast \left|\frac{R_k-{R}_0}{R_0}\right|\ast 100\%$$

Where R_k_ is the adjusted-weight risk map; R_0_ is the initial risk map; N is the number of pixels [15].

The uncertainty map was the standard deviation of the risk maps generated after all factors changed the weights [41]. The weights were randomly selected for each iteration through Monte Carlo sampling (between ± 20%), and the process was repeated 400 times. The total number of output risk maps was calculated by multiplying the number of iterations with the number of criteria. Finally, 400 weight-adjusted values per criteria throughout this range were combined to compute 4000 risk maps [42].

### Risk map validation

The FMD outbreak data in mainland China from 2018 to 2020 were used to test the accuracy of the map. Combining predicted and actual data, the receiver operating characteristic (ROC) analysis was used to assess the predictive ability of the risk map. The R package-InformationValue was used to calculate the ROC.

## Supplementary Information

**Additional file 1 : Supplement 1.** List of risk factors. All factors were assessed by literature and the prevalence of foot-and-mouth disease in China

**Additional file 2 : Supplement 2.** Ten risk factors that were used to calculate the risk mapping.

**Additional file 3 : Supplement 3.** The hierarchical structure of foot-and-mouth disease risk areas assessment.

**Additional file 4 : Supplement 4.** R code of analytic hierarchy process.

## Data Availability

The FMD data came from the Food and Agriculture Organization of the United Nations (http://www.fao.org/home/en/) and the Ministry Agriculture and Rural Affairs of the People's Republic of China (http://www.moa.gov.cn/gk/yjgl_1/yqfb/). The other datasets supporting the conclusions of this study are available in the supplementary materials.

## Abbreviations

FMD: Foot -and -mouth disease
MCDA: Multi-criteria decision analysis
AHP: Analytic hierarchy process
ROC: Receiver operating characteristic
WLC: Weighted linear combination
OAT: One-at-a-time
OIE: World Organization for Animal Health
KDE: Kernel density estimation
MACRs: Mean of absolute change rates
AUC: Area under the ROC curve
