# Supplementary material for: Assessment of foot-and-mouth disease risk areas in mainland China based spatial multi-criteria decision analysis
Source: BMC Vet Res. 2021 Dec 6;17:374. doi: 10.1186/s12917-021-03084-5 (PMC8647368; doi:10.1186/s12917-021-03084-5)
Supplement: Supplementary file 1 — Additional file 1 : Supplement 1. List of risk factors. All factors were assessed by literature and the prevalence of foot-and-mouth disease in China [file 12917_2021_3084_MOESM1_ESM.docx]

**Supplement 1:**

**List of risk factors. All factors were assessed by literature and the prevalence of foot-and-mouth disease in China.**

| Spatial risk factors | Reference(s) |
| --- | --- |
| Human population density (persons/km2) | Chhetri et al. (2010) |
| Buffalo density (buffaloes/km2) | Chhetri et al. (2010) |
| Number of technicians | Chhetri et al. (2010) |
| Number of animals slaughtered daily | Chhetri et al. (2010) |
| Urban population (%) | Chhetri et al. (2010) |
| Number of veterinarians | Chhetri et al. (2010) |
| Number of village animal health workers | Chhetri et al. (2010) |
| Cattle density (cattle/km2) | Chhetri et al. (2010) |
| Number of cattle | Chhetri et al. (2010) |
| Road density (km road/km2) | Chhetri et al. (2010) |
| Goat density | Chhetri et al. (2010) |
| High-frequency movements of cattle | Di Nardo et al. (2011) |
| Multiple farms infection within 3 kilometers | Elnekave et al. (2016a) |
| Distance up to 5 km from neighboring countries | Elnekave et al. (2016b) |
| driest quarter | Jiang et al. (2020) |
| temperature seasonality | Jiang et al. (2020) |
| annual mean temperature | Jiang et al. (2020) |
| precipitation in the driest month | Jiang et al. (2020) |
| mixed farming | Udahemuka et al. (2020) |
| natural breeding | Udahemuka et al. (2020) |
| animals leave shed during day | Wajid et al. (2020) |
| farms, where neighboring farmers used to visit the premises | Wajid et al. (2020) |
| distance to the nearest major international border crossing | Hamoonga et al. (2014) |
| distance to the nearest major road | Hamoonga et al. (2014) |
| Low wetness index | Hamoonga et al. (2014) |
| Median elevation less than 100 m | Hamoonga et al. (2014) |
| purchasing of a new cow without following quarantine protocol | Sansamur et al. (2020) |
| farms located near shared cattle grazing areas in a 10 km radius | Sansamur et al. (2020) |
| FMD vaccination administration by non-official livestock personnel | Sansamur et al. (2020) |
| farms located in a 5 km radius of cattle abattoirs | Sansamur et al. (2020) |
| no history of FMD outbreaks over the previous 12 months in districts where farms were located | Sansamur et al. (2020) |
| Distance from national park or wildlife sanctuary | Molla et al. (2010) |
| Distance up to 20 kilometers from slaughterhouse | Lindholm et al. (2007) |
| Distance up to 20 km from livestock market | Lindholm et al. (2007) |
| Pig density | Hayama et al. (2016) |
| Distance to the outbreak point | Bessell et al. (2010) |
| Serotypes of FMD | Self assessment |
| Density of wildlife | Self assessment |
| Density of sheep | Self assessment |
| Cimatic conditions | Self assessment |
| Movement of animals with FMD | Self assessment |
| The poor living environment of animals | Self assessment |
| Vaccination history | Self assessment |
| Distance to farm | Self assessment |
| Distance to animal quarantine station | Self assessment |
| Distance to water system and wetland | Self assessment |
| Different kinds of animals are raised together | Self assessment |
| The flow frequency of vehicles in the farm | Self assessment |
| Sensitivity for clinical inspection | Self assessment |
| Sex, weight and mental state of animals | Self assessment |

**References**

Chhetri, B.K., Perez, A.M., Thurmond, M.C., 2010. Factors associated with spatial clustering of foot-and-mouth disease in Nepal. Trop. Anim. Health Prod. 42, 1441–1449.

Di Nardo, A., Knowles, N.J., Paton, D.J., 2011. Combining livestock trade patterns with phylogenetics to help understand the spread of foot and mouth disease in sub-Saharan Africa, the Middle East and Southeast Asia. Rev. Sci. Tech. 30, 63–85.

Elnekave, E., van Maanen, K., Shilo, H., Gelman, B., Storm, N., Abed El Khaliq, M., Sharir, B., Berke, O., Klement, E., 2016a. Prevalence and risk factors for foot and mouth disease infection in cattle in Israel. Prev. Vet. Med. 130, 51–59.

Elnekave E , Maanen K V , Shilo H , Gelman B, Storm N, Berdenstain S, Berke O, Klement E., 2016b. Prevalence and risk factors for foot and mouth disease infection in small ruminants in Israel. Prev. Vet. Med. 125, 82–88.

Udahemuka J C , Aboge G O , Obiero G O ., 2020. Risk factors for the incursion, spread and persistence of the foot and mouth disease virus in Eastern Rwanda. BMC Veterinary Research, 16(387),1-10.

Jiang F , Song P , Zhang J ., et al 2020. Assessing the impact of climate change on the spatio-temporal distribution of foot-and-mouth disease risk for elephants. Global Ecology and Conservation, 23:e01176.

Wajid, A., Chaudhry, M., Rashid, H. B., Gill, S. S., & Halim, S. R,. 2020. Outbreak investigation of foot and mouth disease in Nangarhar province of war-torn Afghanistan, 2014. Scientific reports, 10(1), 1-10.

Hamoonga, R., Stevenson, M.A., Allepuz, A., Carpenter, T.E., Sinkala, Y., 2014. Risk factors for foot-and-mouth disease in Zambia, 1981-2012. Prev. Vet. Med. 114, 64–71.

Sansamur C , Arjkumpa O , Charoenpanyanet A , et al. Determination of Risk Factors Associated with Foot and Mouth Disease Outbreaks in Dairy Farms in Chiang Mai Province, Northern Thailand[J]. Animals : an Open Access Journal from MDPI, 2020, 10(3).

Molla, B., Ayelet, G., Asfaw, Y., Jibril, Y., Ganga, G., Gelaye, E., 2010. Epidemiological study on foot-and-mouth disease in cattle: seroprevalence and risk factor assessment in South Omo zone, south-western Ethiopia. Transbound Emerg. Dis. 57, 340–347.

Lindholm, A., Hewitt, E., Torres, P., Lasso, M., Echeverria, C., Shaw, J., Hernandez, J., 2007. Epidemiologic aspects of a foot-and-mouth disease epidemic in cattle in Ecuador. Int. J. Appl. Res. Vet. M. 5, 17–24.

Hayama, Y., Yamamoto, T., Kobayashi, S., Muroga, N., Tsutsui, T., 2016. Potential impact of species and livestock density on the epidemic size and effectiveness of control measures for foot-and-mouth disease in Japan. J. Vet. Med. Sci. 78, 13–22.

Bessell, P.R., Shaw, D.J., Savill, N.J., Woolhouse, M.E., 2010a. Statistical modeling of holding level susceptibility to infection during the 2001 foot and mouth disease epidemic in Great Britain. Int. J. Infect. Dis. 14, e210–215
