# Supplementary material for: Assessment of foot-and-mouth disease risk areas in mainland China based spatial multi-criteria decision analysis
Source: BMC Vet Res. 2021 Dec 6;17:374. doi: 10.1186/s12917-021-03084-5 (PMC8647368; doi:10.1186/s12917-021-03084-5)
Supplement: Supplementary file 2 — Additional file 2 : Supplement 2. Ten risk factors that were used to calculate the risk mapping. [file 12917_2021_3084_MOESM2_ESM.docx]

**Supplement 2:**

**Ten risk factors that were used to calculate the risk mapping.**


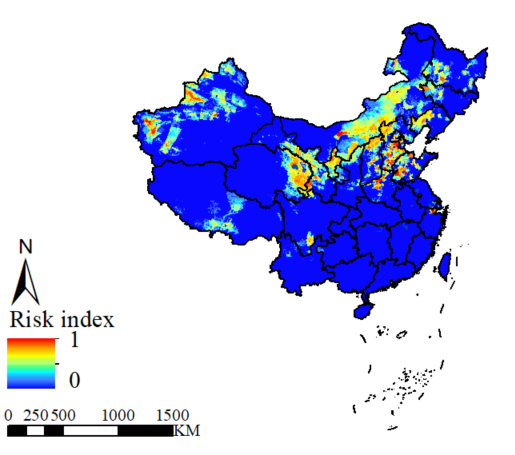


**Fig 1.** Map of sheep density with standardization.


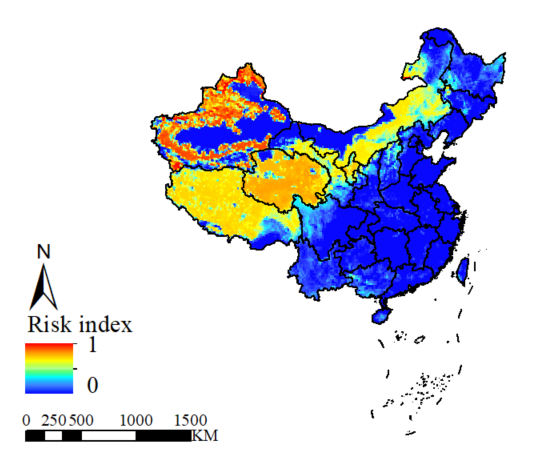


**Fig 2.** Map of rangeland with standardization.


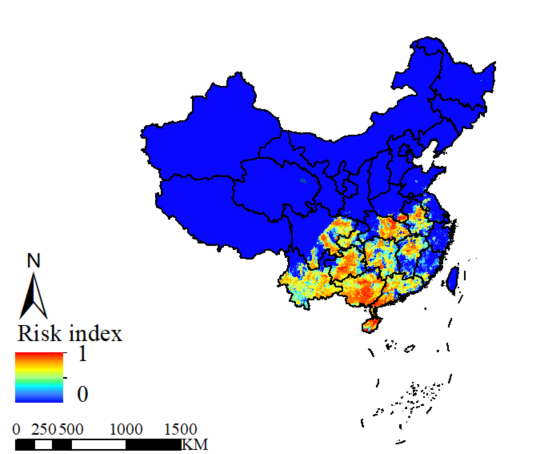


**Fig 3.** Map of buffalo density with standardization.


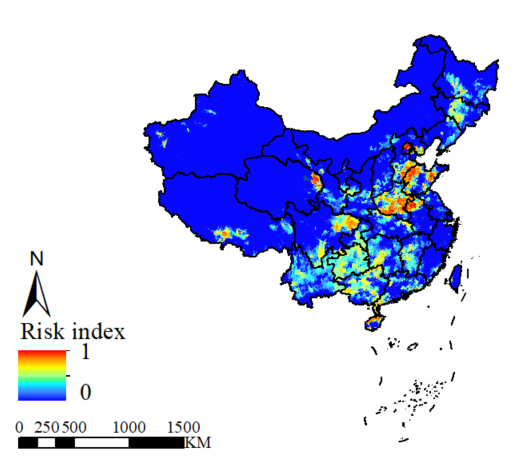


**Fig 4.** Map of cattle density with standardization.


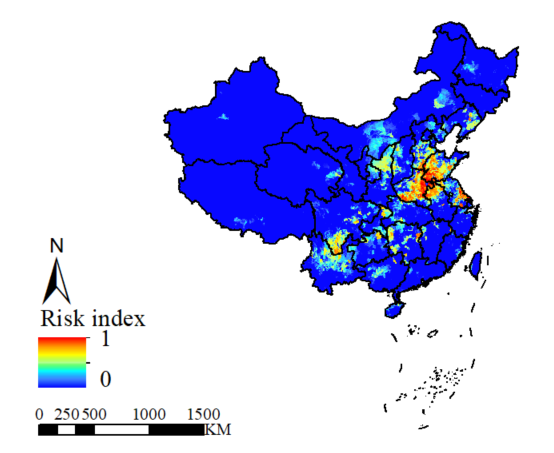


**Fig 5.** Map of goat density with standardization.


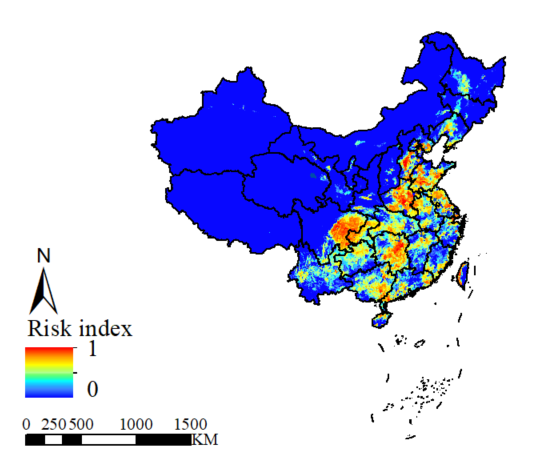


**Fig 6.** Map of pig density with standardization.


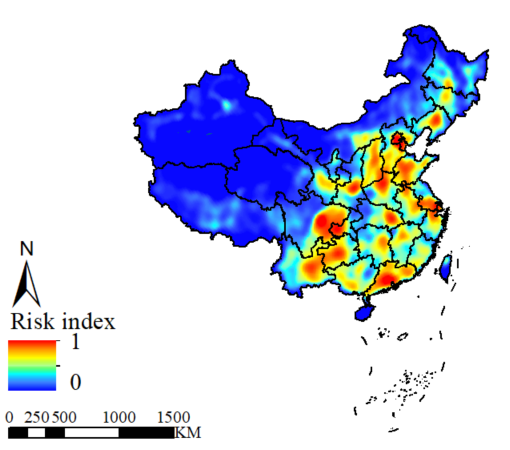


**Fig 7.** Map of major road density with standardization.


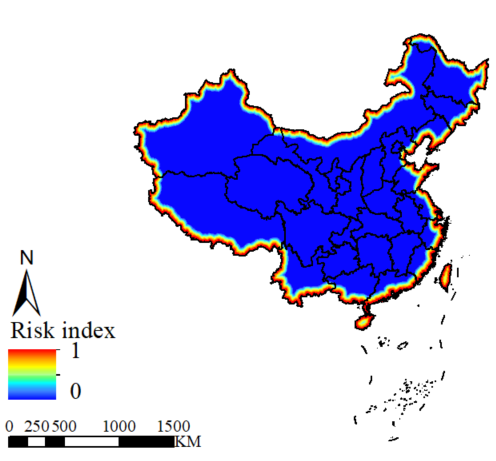


**Fig 8.** Map of distance to national boundaries with standardization.


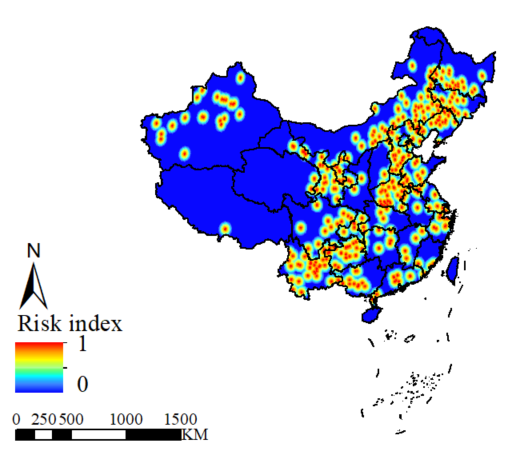


**Fig 9.** Map of distance to livestock market and slaughterhouse with standardization.


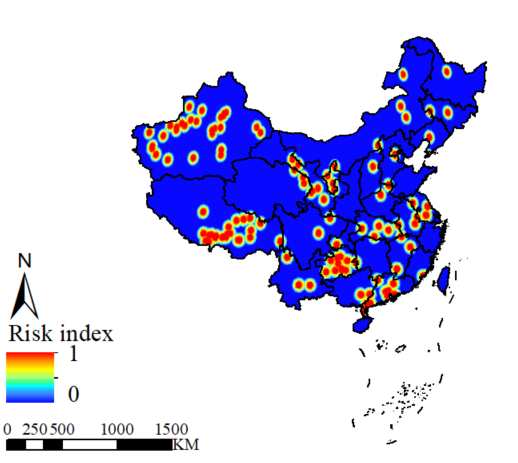


**Fig 10.** Map of distance to previous outbreak points with standardization.
