# Supplementary material for: Assessment of foot-and-mouth disease risk areas in mainland China based spatial multi-criteria decision analysis
Source: BMC Vet Res. 2021 Dec 6;17:374. doi: 10.1186/s12917-021-03084-5 (PMC8647368; doi:10.1186/s12917-021-03084-5)
Supplement: Supplementary file 3 — Additional file 3 : Supplement 3. The hierarchical structure of foot-and-mouth disease risk areas assessment. [file 12917_2021_3084_MOESM3_ESM.docx]

**Supplement 3:**

**The hierarchical structure of foot-and-mouth disease risk areas assessment.**


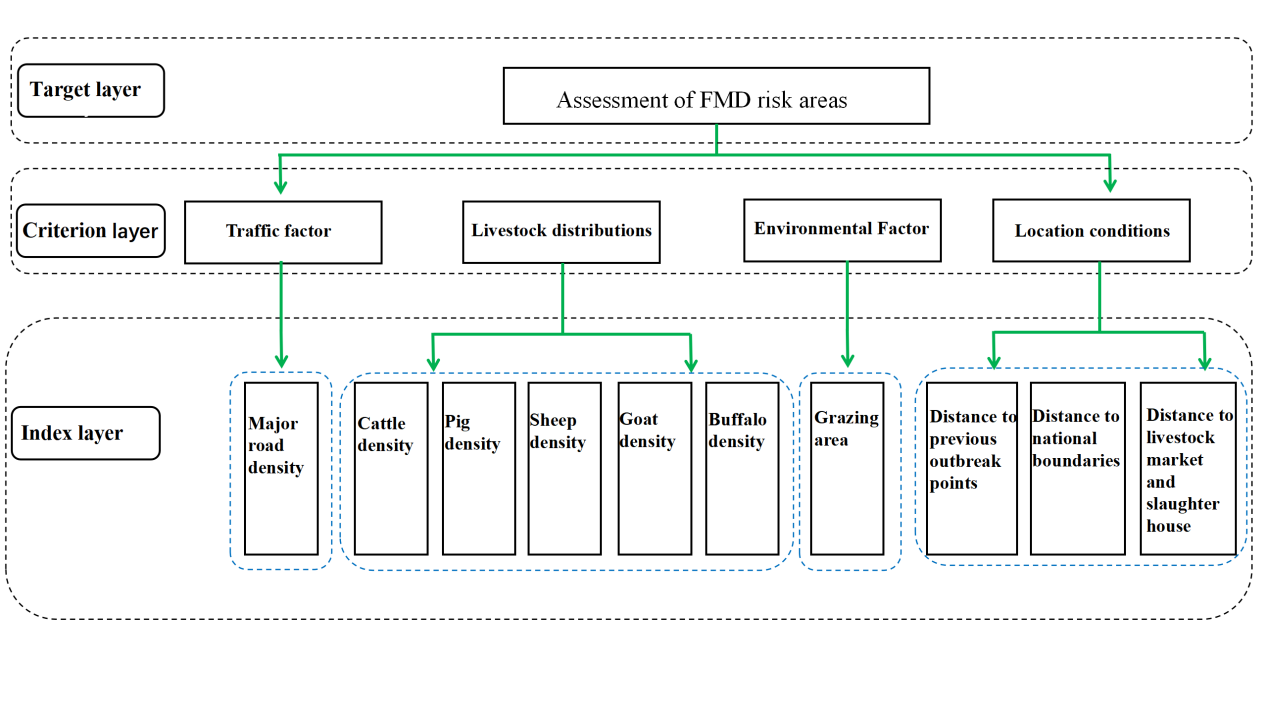


| Target layer (Z) | Criterion layer(A) | The weight of A relative to Z | Index layer(B) | The weight of B relative to A | The weight of B relative to Z |
| --- | --- | --- | --- | --- | --- |
| Assessment of  FMD risk areas | Traffic factor | 0.06 | Major road density | 1 | 0.06 |
|  | Livestock distributions | 0.44 | Cattle density | 0.36 | 0.16 |
|  |  |  | Pig density | 0.18 | 0.08 |
|  |  |  | Sheep density | 0.09 | 0.04 |
|  |  |  | Goat density | 0.09 | 0.04 |
|  |  |  | Buffalo density | 0.27 | 0.12 |
|  | Environmental  factor | 0.14 | Grazing area | 1 | 0.14 |
|  | Location conditions | 0.36 | Distance to previous outbreak points | 0.5 | 0.18 |
|  |  |  | Distance to national boundaries | 0.22 | 0.08 |
|  |  |  | Distance to livestock market  and slaughterhouse | 0.28 | 0.10 |
