# Supplementary material for: Assessment of foot-and-mouth disease risk areas in mainland China based spatial multi-criteria decision analysis
Source: BMC Vet Res. 2021 Dec 6;17:374. doi: 10.1186/s12917-021-03084-5 (PMC8647368; doi:10.1186/s12917-021-03084-5)
Supplement: Supplementary file 4 — Additional file 4 : Supplement 4. R code of analytic hierarchy process. [file 12917_2021_3084_MOESM4_ESM.docx]

**Supplement 4:**

**R code of analytic hierarchy process.**

######whr0458############

rm(list=ls())

gc()

library("readxl")

library("dplyr")

library("magrittr")

matrix_c <- as.matrix(a)

matrix_c <-

if(class(data) == 'matrix'){

data = data

} else {

if ( class(data) == 'data.frame' & nrow(data) == ncol(data) - 1 & is.character(data[,1,drop = TRUE])){

data = as.matrix(data[,-1])

} else if (class(data) == 'data.frame' & nrow(data) == ncol(data)) {

data = as.matrix(data)

} else {

stop('please recheck your data structure , you must keep a equal num of the row and col')

}

}

sum_vector_row = data %>% apply(2,sum)

decide_matrix = data %>% apply(1,function(x) x/sum_vector_row)

weigth_vector = decide_matrix %>% apply(2,sum)

result = list(decide_matrix = decide_matrix, weigth_vector = weigth_vector/sum(weigth_vector ))

return(result)

}

Weigth_fun(matrix_c)

################

AW_Weight <- function(data){

if(class(data) == 'matrix'){

data = data

} else {

if ( class(data) == 'data.frame' & nrow(data) == ncol(data) - 1 & is.character(data[,1,drop = TRUE])){

data = as.matrix(data[,-1])

} else if (class(data) == 'data.frame' & nrow(data) == ncol(data)) {

data = as.matrix(data)

} else {

stop('please recheck your data structure , you must keep a equal num of the row and col')

}

}

AW_Vector = data %*% Weigth_fun(data)$weigth_vector

λ = (AW_Vector/Weigth_fun(data)$weigth_vector) %>% sum(.) %>% `/`(length(AW_Vector))

result = list(

AW_Vector <- AW_Vector,

`∑AW/W`<- AW_Vector/Weigth_fun(data)$weigth_vector,

λ <- λ)

return(result)

}

AW_Weight(matrix_c)

#######Consist_Test ##########

Consist_Test <- function(λ,n){

RI_refer = c(0,0,0.52,0.89,1.12,1.26,1.36,1.41,1.46,1.49,1.52,1.54)

# RI_refer = c(0,0,0.52,0.89,1.12,1.26,1.36,1.41,1.46,1.49,1.52,1.54,1.56,1.58,1.59)

CI = (λ - n)/(n - 1)

CR = CI/(RI_refer[n])

if (CR <= 0.1){

cat(" 通过一致性检验！",sep = "\n")

cat(" Wi: ", round(CR,4), "\n")

} else {

cat(" 请调整判断矩阵！","\n")

}

return(CR)

}

Consist_Test(AW_Weight(matrix_c)[[3]],10)
